# Nerve loop around the axillary vessels by the roots of the median nerve a rare variation in a south Indian male cadaver: a case report

**DOI:** 10.1186/1757-1626-2-179

**Published:** 2009-10-31

**Authors:** Kumar MR Bhat, Siddaraju Gowda, Bhagath Kumar Potu

**Affiliations:** 1 Department of Anatomy, Kasturba Medical Collage, Manipal University, Manipal-576104, India

## Abstract

**Introduction:**

Median nerve is normally formed by the union of medial and lateral root arising from the medial and the lateral cords of the brachial plexus respectively. However, variations in the formation and its relation with the axillary vessels are not uncommon. Therefore, knowledge of the variations in the nerve formation and course is useful for the clinicians during surgery and for differential diagnosis of uncommon clinical conditions.

**Case presentation:**

During the routine dissection in the department of anatomy, Kasturba Medical Collage, Manipal, India, we found unique anatomical variations in the formation and the course of the roots of the median nerve forming the neural loops around the axillary artery and vein.

**Conclusion:**

Here we report the detailed description of these variations along with its clinical, embryological relevance and review of literature.

## Introduction

The brachial plexus is formed normally by the fusion of the anterior primary rami of C5-8 and T1 spinal nerves and supplies the muscles of the back and the upper limb. The C5 and C6 roots fuse to form the upper trunk, the C7 continues as the middle trunk and the C8 and T1 join to form the lower trunk. Each trunk further divides into anterior and posterior divisions. The anterior divisions of the upper and middle trunks form the lateral cord, the anterior division of the lower trunk continues as the medial cord and the posterior divisions of all three roots form the posterior cord and supply the upper limb. The median nerve is usually formed by two roots, the lateral (C5, 6, 7) and medial (C8, T1) roots arising from the lateral and medial cords respectively. These roots embrace the third part of the axillary artery, and join together anterior or lateral to the axillary artery to form the median nerve [1]. Due to the complex nature of development of brachial plexus and axillary vessels, the formation, branching pattern of nerves and their relation to axillary vessels may show many anatomical variations [2-4]. The two roots of the median nerve may enclose the axillary vein as well as the axillary artery. The median nerve or its roots may pass behind the axillary artery instead of in front of it [5]. In this present study, we describe a rare and unreported variation in the formation of the median nerve and its relation to axillary vessels.

## Case presentation

**Figure 1:**
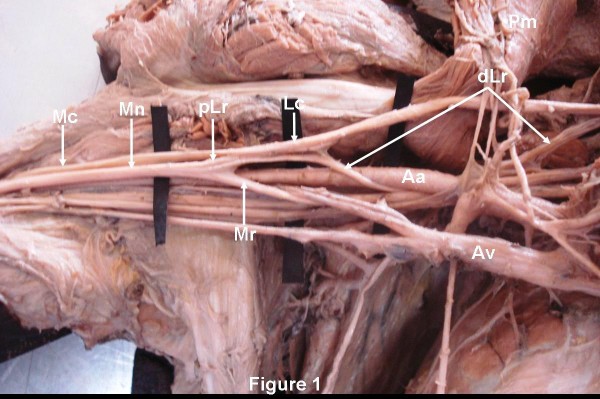
**The Lateral root of the median nerve (Mn) had two parts**. The distal part of the lateral root (dLr) was arising from the lateral cord (Lcd) of the brachial plexus medial to pectoralis minor muscle (Pm) and winds round the axillary artery (Aa) before joining the lateral cord again. The proximal part of the lateral root (pLr) was arising from lateral cord lateral to pectoralis minor muscle and joins the medial root (Mr) of median nerve. The medial root of median nerve (Mn) was arising from medial cord (Mcd) and then winds round the axillary vein (Av) before joining the lateral root to form median nerve. Mc- Musculocutaneous nerve.

**Figure 2:**
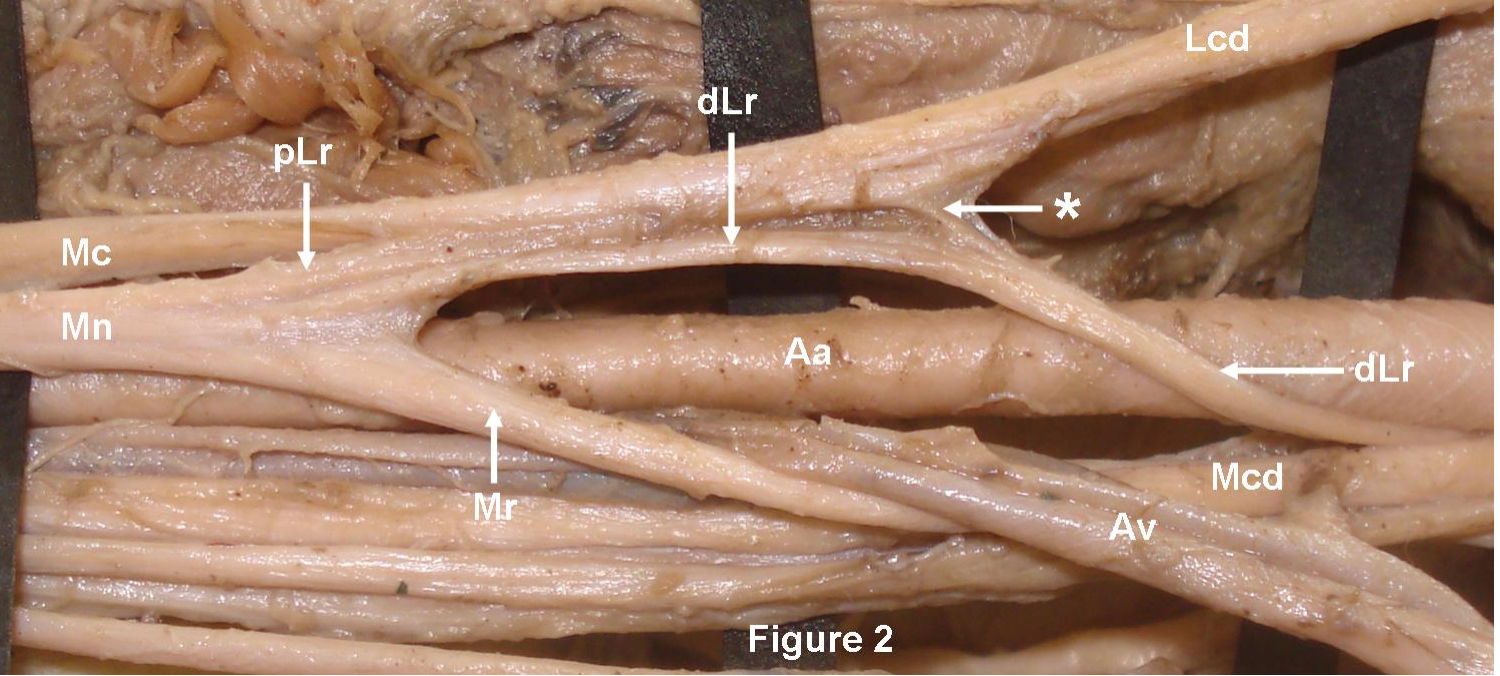
**The distal part of the lateral root (dLr) after winding round the axillary artery (Aa), majority of the fibers were just connected to lateral cord (Lcd) of the brachial plexus by connective tissue and then deviates with proximal part of the lateral root (pLr) to form median nerve (Mn)**. Few fibers of the distal part of the lateral root blend with fibers of the lateral cord and emerge along with proximal part of the lateral root. Mcd-Medial cord of the brachial plexus; Mc- Musculocutaneous nerve. * Few fibers of the distal part of the lateral root.

During the routine dissection (36 cadavers) in the department of Anatomy, Kasturba Medical College, Manipal, India, in the right axilla of a 58-year-old South Asian (Indian) male cadaver, the median nerve was formed by the two roots, lateral and medial as normally described. However, the lateral root of the median nerve was arising much earlier than its usual site. The lateral root was arising from the lateral cord, just after the formation of the lateral cord, proximal to pectoralis minor muscle, posterolateral to the axillary artery. Then, fibers of the lateral root (distal part) runs along with the medial cord of the brachial plexus winding round the axillary artery from posterolateral to medial side to join the lateral cord again distal to pectoralis major muscle. Thus, forming a nerve loop around axillary artery in posterolateral to anteromedial direction (Figure 1). While joining the lateral cord, majority of the lateral root fibers were just connected to the lateral cord by connective tissue septum and then leaves the lateral cord (proximal part) to join the medial root of the median nerve. However, few fibers of the lateral root were actually blended with the lateral cord and again directed back to the lateral root of the median nerve (Figure 2). The lateral root of the median nerve was joining the medial root distal to pectoralis minor muscle. The medial root had its normal single origin from the medial cord of the brachial plexus, but it was winding round the axillary vein lateral to the pectoralis minor muscle before joining the lateral root. Thus, another neural loop of the lateral and medial root of the median nerve was formed around the axillary vein (Figure 1). Finally, the median nerve thus formed runs anterior to the third part of the axillary artery. However, the course and the origin of the lateral and medial roots of the median nerve was normal in the left axilla.

## Discussion

Earlier, Pandey and his group found, an abnormal formation and course of the median nerve in 7% of the cadavers. They found that, the medial root received the communicating branch/branches either from the lateral or posterior cord; both roots of the median nerve were joined on medial side of the axillary artery to form the median nerve. Roots of the median nerve did not join to form the median nerve in the axilla, but, both traveled separately anteromedial to the axillary and brachial arteries [6]. In another study, the median nerve was shown to formed by the fusion of three branches; two from the lateral cord, and one from the medial cord. The abnormal root coming from the lateral cord had very close oblique course over the axillary artery [7]. It has also been shown that, the lateral cord of the brachial plexus after piercing the coracobrachialis and after giving branches to the muscles of the front of the arm, gave one branch as lateral root of the median nerve at the level of the middle of the arm. Later this root joined the medial root to form the median nerve [8]. Goyal and his co-worker found the median nerve to be formed by three roots; the additional root was from the anterior division of the middle trunk [9]. Uzun and his team found median nerve to be formed by four branches, three of them arising from the lateral cord and one from the medial cord [10]. Interestingly, in another study, additional root in the formation of the median nerve was found in 52% of the cases. In 4 cases one of the two lateral roots came from the anterior division of the middle trunk and one from the lateral cord. In 24 cases they found two lateral roots coming from the lateral cord [11]. The lateral and medial roots of the median nerve found to joining in the distal part of the arm rather in the axilla itself [11,12] and median nerve is also found to fuse with musculocutaneous nerve in the axilla [13].

Many variations in the relationship of the axillary artery with the roots of the median nerve have been reported. The lateral root of the median nerve found to cross the axillary artery anteriorly and meets the medial root such that the median nerve lies medial to the third part of axillary artery [3]. Das and Paul have observed a similar case where there were two lateral roots of the median nerve [14].

The subclavian and the axillary system of arteries are derived from the seventh cervical intersegmental artery [15]. Hence, the artery passes between the lateral and the medial cords, representing the fifth, sixth and seventh cervical nerve on the one side and the eighth cervical and first thoracic nerves on the other side. Further, the artery may also arise from the sixth, the eighth or the ninth intersegmental artery leading to abnormal relations with the brachial plexus [4]. Further, the plexus is in turn may modified by the presence of the abnormally placed artery. The segregation of developing structures within the limb bud has a directive action upon the growing nerve fibers and subsequently grouping into definite bundles. The presence of anomalies may be attributed to random factors influencing the mechanism of formation of the limb muscles and the peripheral nerves during embryonic life [2]. Brachial plexus is a routing system to get nerves with common function into the proper terminal nerves. Therefore, errors in the pattern of distribution in the braches of the brachial plexus may finally be corrected distally in the arm, forearm or in the hand before their target innervations [16].

The knowledge of the variations of the peripheral nervous system is useful in clinical as well as surgical practice as they may be the cause of nerve palsy syndromes, ischemia because of variable relations of nerves with the surrounding muscles and the vessels. The presence of anatomical variations in the peripheral nerves is used to explain unexpected signs and symptoms useful in medical practice. Owing to more reliance on computer imaging in the diagnostic medicine, variations of the brachial plexus are vulnerable to injury during the routine surgical procedures of the axilla [17] and the upper arm, such as nerve block, cannulation of the axillary vessels during cardiopulmonary bypass [18], axillary artery perfusion, while transposing and anastomosing the cephalic vein with axillary vein to treat cephalic arch stenosis [19], internal fixation of humeral fracture, surgical resection of axillary tumors, repair of shoulder dislocation, vessels and nerve repair after trauma, radical mastectomy, lymph node biopsy, lymphodectomy of axilla etc. Anomalous branches and the course of the nerves crossing the axillary artery anteriorly may cause compression syndromes producing ischemia. Further, the knowledge of the possible variations in the brachial plexus seems to be very useful while assessing the same via MRI [20]. Thus, these variations are important to note by the anatomist, clinicians and also by the radiologist.

## Abbreviations

Mn: Median nerve; dLr: Distal part of the lateral root; pLr: Proximal part of the lateral root; Lc: Lateral cord of the brachial plexus; Mr: Medial root: Pm: Pectoralis minor muscle; Aa: Axillary artery; Av: Axillary vein; Mc: Musculocutaneous nerve; Mcd: Medial cord of the brachial plexus; *: Few fibers of the distal part of the lateral root.

## Consent

Written informed consent was obtained from the concerned authorities for publication of this case report. A copy of the written consent is available for review by the Editor-in-Chief of this journal.

## Competing interests

The authors declare that they have no competing interests.

## Authors' contributions

SG found the variation, KB took photographs and prepared the manuscript and BP- assisted in preparation of manuscript. All authors read and approved the final manuscript.

## References

[B1] Standring S Gray's Anatomy 2005 39 Edinburgh: Elsevier/Churchill Livingstone 846 848

[B2] Streeter GL Keibel F, Mall FP From The development of nervous system: III. Peripheral nervous system Handbook of human embryology 1912 2 Philadelphia: JB Lippincott Co 1 156

[B3] Singhal S Rao VV Ravindranath R Variations in brachial plexus and the relationship of median nerve with the axillary artery: a case report J Brachial Plex Peripher Nerve Inj 2007 3 2 21 10.1186/1749-7221-2-21 PMC2082021 17915015

[B4] Yang HJ Gil YC Lee HY Intersegmental Origin of the Axillary Artery and Accompanying Variation in the Brachial Plexus Clin Anat 2009 22 586 594 10.1002/ca.20811 19484799

[B5] Bergman RA Thompson SA Afifi AK Saadeh FA Compendium of human anatomic variations Urban & Schwarzenberg, Baltimore-Munich 1988

[B6] Pandey SK Shukla VK Anatomical variations of the cords of brachial plexus and the median nerve Clin Anat 2007 20 150 156 10.1002/ca.20365 16795062

[B7] Sargon MF Uslu SS Celik HH Akşit D A variation of the median nerve at the level of brachial plexus Bull Assoc Anat (Nancy) 1995 79 246 25 26 8541607

[B8] Le Minor JM A rare variation of the median and musculocutaneous nerves in man Arch Anat Histol Embryol 1990 73 33 42 1669679

[B9] Goyal N Harjeet Gupta M Bilateral variant contribution in the formation of median nerve Surg Radiol Anat 2005 8 1 4 10.1007/s00276-005-0023-6 16151971

[B10] Uzun A Selling LL A variation in the formation of median nerve and communicating branches between musculocutaneous and median nerve Folia Morphol (Warsz) 2001 60 2 99 101 11407150

[B11] Sassoli Fazan VP Amadeu AS Caleffi AL Rodrigues Filho OA Brachial plexus variation in its formation and main branches Acta Cir Bras 2003 18 5

[B12] Uysal II Seker M Karabulut AK Buyukmumcu M Ziylan T Brachial plexus variations in human fetuses Neurosurgery 2003 3 676 684 10.1227/01.NEU.0000079485.24016.70 12943583

[B13] Salopek D Dujmovic A Hadjina J Topic I Bilateral arterial and nervous variations in the human upper limb: a case report Ann Anat 2007 189 3 290 294 10.1016/j.aanat.2006.09.007 17534038

[B14] Das S Paul S Anomalous branching pattern of lateral cord of brachial plexus Int J Morpho 2005 23 289 292

[B15] Wollard HH The development of the principal arterial stems in the forelimb of the pig. Contrib Embryol 1922 14 139

[B16] Tountas CP Bergman RA Anatomic variations of the upper extremity 1993 New York: Churchill Livingstone 195 203

[B17] Retzl G Kapral S Greher M Mauritz W Ultrasonographic findings of the axillary part of the brachial plexus Anesth Analg 2001 92 5 1271 1275 10.1097/00000539-200105000-00037 11323361

[B18] Zattera G Totaro P D'Armini AM Vigano M Deltoido-pectoralis approach to axillary vessels for full-flow cardiopulmonary bypass Eur J Cardiothorac Surg 2009 35 5 913 914 10.1016/j.ejcts.2009.01.049 19318271

[B19] Kian K Unger SW Mishler R Schon D Lenz O Asif A Role of surgical intervention for cephalic arch stenosis in the "fistula first" era Semin Dial 2008 21 1 93 96 10.1111/j.1525-139X.2007.00388.x 18034783

[B20] Van Hoof T Mabilde C Leybaert L Verstraete K D'Herde K Technical note: the design of a stereotactic frame for direct MRI-anatomical correlation of the brachial plexus Surg Radiol Anat 2005 27 6 548 556 10.1007/s00276-005-0049-9 16249823

